# Mobility, Physical Activity, and Social Engagement of Community-Living Older Adults

**DOI:** 10.1093/geroni/igab046.096

**Published:** 2021-12-17

**Authors:** Wenjun Li, Lien Quach

## Abstract

Mobility, physical activity and social engagement are important to healthy aging and independent living among older adults. This symposium includes four related studies on these issues. Dr. Lien Quach and her team examined racial and ethnic disparities in social engagement among community-living older adults using data from the national Health and Retirement Study. The analysis found that Asians and Hispanics had significantly lower social engagement score compared with non-Hispanic Whites, advocating for further investigations of the causes of racial disparities in social engagement. Dr. Su-I Hou’s study examined the impact of physical activity and social relationship on social engagement. The study found positive impacts of more physical activity, better social relationships and volunteers on social engagement. The results have important implications to promotion of social engagement among older adults participating in aging-in-community programs. Dr. Ladda Thiamwong’s study demonstrated the benefits of using assistive health technology (AHT) to assess the relationships between fall risks, body compositions and objectively measured physical activity in older adults. Dr. Thiamwong’ will discuss the research protocol and preliminary results. Dr. Li’s Health Aging and Neighborhood Study examined variations of older adults’ driving behaviors by sex, age, race, income, health status and housing density of the neighborhoods. The study found substantial differences in mobility and driving patterns by both personal characteristics and neighborhood living environment. The findings have important implications to community programs that support older adults aging in place.

